# Neurological Manifestations of COVID-19: Causality or Coincidence?

**DOI:** 10.14336/AD.2020.0917

**Published:** 2021-02-01

**Authors:** Fangfang Zhao, Ziping Han, Rongliang Wang, Yumin Luo

**Affiliations:** ^1^Institute of Cerebrovascular Disease Research and Department of Neurology, Xuanwu Hospital of Capital Medical University, Beijing, China.; ^2^Beijing Geriatric Medical Research Center and Beijing Key Laboratory of Translational Medicine for Cerebrovascular Diseases, Beijing, China.; ^3^Beijing Institute for Brain Disorders, Capital Medical University, Beijing, China.

**Keywords:** SARS-CoV-2, COVID-19, neurological manifestations, nerve invasion

## Abstract

The COVID-19 pandemic that swept the world at the beginning of 2020 is still raging. It is well established that in addition to respiratory symptoms, COVID-19 can also have neurological manifestations that may result from direct or indirect neurological damage. But are these neurological manifestations coincidental or causal? From a neurological perspective, these symptoms could be the result of neurological damage following SARS-CoV-2 infection, or they could be coincidental, from causes such as secondary systemic complications or side effects of drug treatment. The aim of this review is to raise clinician’s awareness to the development of neurological impairment in SARS-CoV-2 infected patients in the current normative prevention and control.

In the past ten months, COVID-19, formerly known as 2019 novel coronavirus, has become a serious threat to global health. In March, the World Health Organization (WHO) increased the characterization from epidemic to global pandemic. More than 31.3 million people worldwide are currently affected by the disease, and more than 962,092 have already died as a result. The coronaviruses (CoVs) are a large group of positive-strand RNA viruses which infect Vertebrata in the natural world [1]. The coronavirus strain responsible for causing COVID-19 belongs to the genus *Betacoronavirus* and can be transmitted from person to person [2]. Seven viruses belonging to the genus *Betacoronavirus* are known to cause human respiratory tract infection and transmission, causing diseases such as Middle East respiratory syndrome (MERS) and severe acute respiratory syndrome coronavirus (SARS-CoV) [3]. Chinese scientists first isolated the circulating coronavirus strain on January 7, 2020, and shortly after, on February 11, the associated disease was officially named by the WHO as coronavirus disease 2019 (COVID-19) [4]. The novel CoV was named SARS-CoV-2.

It is well established that CoVs can have neurological manifestations resulting from direct or indirect neurological damage [5,6] in addition to respiratory symptoms. However, are neurological manifestations of COVID-19 coincidental or causal? The neurological effects include specific symptoms such as stroke and disorders of smell and taste, etc. From a neurological perspective, these symptoms could be the result of neurological damage following SARS-CoV-2 infection, or they could be coincidental, resulting from causes such as secondary systemic complications or side effects of drug treatment. Therefore, based on the existing COVID-19 literature, this paper further discusses the mechanism of neurological damage after SARS-CoV-2 infection in relation to the neurological manifestations that occur in infected patients (Figure 1). The aim is to raise awareness among clinicians about the development of neurological impairments in SARS-CoV-2 infected patients in the current normative prevention and control.

**Figure 1.**
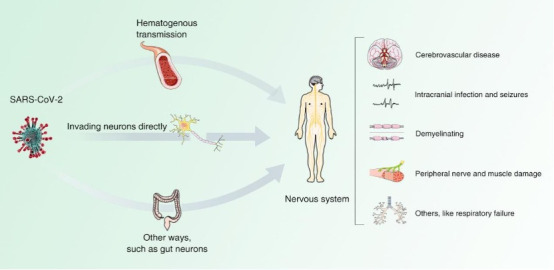
Neurological manifestations and possible mechanisms of neurological impairment in COVID-19.

## Cerebrovascular disease

Stroke associated with COVID-19 was first reported by Mao *et al*. (2020) in China [7]. Hundreds of cases of stroke associated with COVID-19 have since been reported around the world. The prevalence of cerebrovascular disease in COVID-19 patients is high, with the incidence ranging from 0.7-5.1% (Table 1). Merkler *et al.* (2020)found that approximately 1.5% of emergency or hospitalized COVID-19 patients experienced an ischemic stroke, a rate 7.5 times higher than observed in influenza patients [8]. In a study of 214 patients in Wuhan, five were diagnosed with ischemic stroke, and one was diagnosed with hemorrhagic stroke [7]. In addition to this, a number of case reports have described patients with COVID-19 who had stroke as their primary symptom. Of the reported COVID-19 patients with cerebrovascular disease, the majority had ischemic strokes [7,9-15] and a small proportion had hemorrhagic strokes [7, 9, 14]. One of the reasons for the occurrence of cerebral hemorrhage in COVID-19 patients is presumed to be related to thrombocytopenia[16-19]. According to the available reports, cerebrovascular disease occurs more commonly in patients with severe infections. The study by Mao et al. (2020) showed that stroke occurred in 5.7% of patients with severe disease, and 0.8% of patients with mild disease [7]. In the Netherlands, ischemic stroke occurred in 2.7% of critical COVID-19 patients across three hospitals (5 out of 184 patients) [10]. The reasons for the more common occurrence of cerebrovascular disease in critically ill patients may relate to the following: a higher proportion of elderly patients with severe infections [13], higher blood D-dimer levels [7], a hypercoagulable state [20], the inflammatory response [21,22], and in some patients, high blood anti-cardiolipin antibodies [18]. SARS-CoV-2 infection may also cause damage to cerebral blood vessels [14, 23-27]. To summarize, the mechanisms suggested in the literature for the occurrence of cerebrovascular damage in patients with COVID-19 are complex and multifactorial. Prompt diagnosis and the treatment of acute cerebrovascular disease, delivered in conjunction with the treatment of COVID-19, may be key to reducing patient mortality.

From the evidence presented above, it appears possible that the occurrence of cerebrovascular disease alongside COVID-19 is purely coincidental. The high prevalence of cerebrovascular disease among COVID-19 patients does not in itself prove that incidence of cerebrovascular disease in COVID-19 patients is a causal factor. According to the available reports, most patients with COVID-19 combined with cerebrovascular disease have risk factors for cerebrovascular disease, such as hypertension, diabetes, coronary artery disease, hyperlipidemia, atrial fibrillation, and smoking. This is reflected in the proportions of the population with risk factors for ischemic and hemorrhagic stroke, which were ≥50% and ≥75%, respectively (Table 2). The risk of stroke may therefore be increased in a state of infection and systemic inflammation. However, direct damage to the cerebrovascular system caused by SARS-CoV-2 infection cannot be ruled out based on the current literature.

**Table 1:** Summary of the cerebrovascular complications of COVID-19.

Authors	Total Cases	Type of CVD in patients with COVID-19	
		IS - n(%)	CH - n(%)
Mao *et al.* [7]	214	5 (2.3)	1 (0.5)
Helms *et al.* [12]	64	3 (4.7)	
Klok *et al*. [10]	184	5 (2.7)	
Li *et al.* [14]	219	10 (4.6)	1 (0.5)
Annie *et al.* [28]	9358	64 (0.7)	
Rothstein *et al.* [29]	844	20 (2.4)	8 (0.9)
Stéphane *et al.* [30]	64	17 (27)	
Merkle *et al.* [8]	1916	31 (1.6)	
Francisco *et al.* [31]	1683	17 (1.0)	5 (0.3)

CVD: cerebrovascular disease, IS: ischemic stroke,CH: cerebral hemorrhage.

### Demyelinating lesions of the central nervous system

During the COVID-19 pandemic, there have been relatively few reports of central nervous system (CNS) demyelination arising from SARS-CoV-2 infection. A total of five cases have been reported, which are discussed below. Reichard et al. (2020) performed an autopsy on a patient who died of SARS-CoV-2 infection, and neuropathological examination revealed hemorrhagic white matter lesions throughout the cerebral hemisphere, peripheral axonal damage, and a perivascular acute disseminated encephalomyelitis (ADEM)-like appearance [40]. Palao *et al*. (2020) reported a case of multiple sclerosis in a patient who presented on cranial MRI with a right-sided optic nerve inflammation and CNS demyelinating lesions [41]. Zanin *et al*. (2020) [42] from Canada reported a case of new demyelinating lesions around the periventricular and medullary cervical junction of the medulla oblongata. Two other patients presented with acute myelitis with demyelinating changes in the spinal cord [43], and acute necrotizing encephalitis showing hemorrhagic necrosis in the bilateral medial temporal lobes and thalamus of the brain [44]. Most of these cases occurred shortly after SARS-CoV-2 infection. The short time interval led us to consider whether there was a causal relationship between the two.

The development of demyelinating lesions after SARS-CoV-2 infection may be related to the immune response to viral infection. Previous studies have suggested that CoVs cause demyelination of the CNS. Viral infection has been shown to be associated with multiple sclerosis [45,46], and human CoVs were present in autopsies of multiple sclerosis patients [47]. Human CoVs have also been found in the cerebrospinal fluid of children with acute disseminated encephalomyelitis. It is therefore possible that SARS-CoV-2 may be a direct cause of demyelinating lesions in the CNS.

### Encephalopathy and encephalitis

Encephalopathy and encephalitis are serious CNS manifestations of SARS-CoV-2 infection. COVID-19 patients with encephalopathy/encephalitis generally have a poorer prognosis. They are mostly elderly patients [48], who may have a high number of associated comorbid chronic diseases [49] or immune deficiency. Clinical manifestations of encephalopathy or encephalitis include fever, headache, impaired consciousness, and epilepsy [7,50,51], and are visible in MRI as altered cortical and subcortical T2/FLAIR signals. Recently, SARS-CoV-2 virus was detected in the cerebrospinal fluid of two patients [52,53]; the first case was at the Beijing Tiantan Hospital in China, and the second was in Japan. In the latter case, the cranial MRI also showed signs of encephalitis. However, the vast majority of patients with COVID-19-related encephalopathy or encephalitis have normal cerebrospinal fluid reports. While it is possible that the presence of SARS-CoV-2 in cerebrospinal fluid could be due to the virus breaking the blood-brain barrier and entering the CNS, it could also be due to contamination of the cerebrospinal fluid. Therefore, there is still a lack of sufficient evidence to prove that SARS-CoV-2 virus infection directly invades the CNS and then causes encephalopathy or encephalitis. However, the possibility of a causal relationship cannot be discounted entirely.

### Guillain-Barre syndrome (GBS)

To date, there is no evidence that SARS-CoV-2 infection can be triggered or is incidentally associated with GBS. But the possible involvement of COVID-19 in the peripheral nervous system has attracted considerable attention. According to the available reports, approximately 14 cases of COVID-19 combined with GBS [54-64] have been reported, including two cases of Miller-Fisher syndrome [57]. All patients had fever and respiratory symptoms 5-10 days before the onset of neurological symptoms and went on to develop respiratory failure. It is unclear whether the respiratory failure is associated with neuromuscular dysfunction due to GBS or with a previous severe respiratory infection.

Further investigations, including large trials and case-control studies, should therefore be conducted to clarify the linkages and possible causal relationships. While we know that post-infection molecular simulation plays an important role in the development of GBS, this role has only been confirmed in animal models of *Campylobacter jejuni* infection and not in animal models of other viral infections. It therefore may not serve as a mechanism for SARS-CoV-2 associated GBS.

**Table 2:** Characteristics of patients with COVID-19 and stroke.

Authors	N	IS				CH			

		Male, n (%)	Age (yrs)	Risk factors (%)	Number	Male, n (%)	Age (yrs)	Risk factors (%)	Number
Rothstein *et al.* [29]	28	12 (60)	64±12	≥95	20	4(50)	57±7	≥75	8
Katz *et al.* [33]	86	NA	NA	NA	72	NA	NA	NA	14
Francisco *et al.* [31]	22	48 (72.7)	68.2 ± 13	≥58.8	17	4 (80)	62.6 ± 7.2	≥80	5
Escalard *et al.* [34]	10	8 (80)	59.5	≥50	10	NA	NA	NA	NA
Markler *et al.* [32]	31	18 (58)	69	≥97	31	NA	NA	NA	NA
Siddhant *et al.* [35]	33	NA	NA	NA	NA	26 (78.8)	61.6	NA	33
Ying *et al.* [36]	135	81 (62.3)	63.4 ± 13.1	≥64.5	135	NA	NA	NA	NA
George *et al.* [37]	174	108 (62.1)	71.2	≥68.4	174	NA	NA	NA	NA
Li *et al.* [14]	11	5 (50)	73	N/A	10	1 (100)	60	NA	1
Oxley *et al.* [13]	5	4 (80)	40.4	60	5	NA	NA	NA	NA
Avula *et al.* [11]	4	1(25)	81	100	4	NA	NA	NA	NA
Tunç *et al.* [15]	4	2 (50)	65.25	100	4	NA	NA	NA	NA
Nicole *et al.* [38]	4	3 (75)	56	100	4	NA	NA	NA	NA
Mauro *et al.* [39]	6	3 (75)	74.3	100	4	2 (100)	57	50	2

IS:ischemic stroke,CH:cerebral hemorrhage

### Dysomia and dysgeusia

Impairments of the senses of smell and taste are two of the most prevalent symptoms of SARS-CoV-2 infection during epidemics of novel coronavirus pneumonia. Approximately 70% of patients appear to have a reduced sense of smell and taste during the course of the disease [65,66]. Chinese scholars have reported that of 214 individuals with COVID-19, there were 12 cases of impaired taste, 11 cases of impaired smell, and 3 cases of visual impairment [7]. In Europe, a multicenter study of 417 patients reported 357 cases of olfactory impairment, 342 cases of taste dysfunction, and 11.8% cases of olfactory dysfunction preceding other symptoms [65]. Some patients have also been reported with the onset of transient loss of smell and taste [64]. A curious phenomenon has also been found in some patients where if one family member had olfactory or gustatory impairment, other family members had similar symptoms for a short period [67].

If the relationship between SARS-CoV-2 infection and neurological damage is causal, then impairments of the senses of smell and taste are the strongest evidence. The primary reason is that in a study of olfactory dysfunction in patients with viral infection, Suzuki et al. (2007) showed that some patients had normal nasal acoustic reflex measurements but still had olfactory deficits [68]. This suggests that nasal inflammation and nasal obstruction are not the only causes of olfactory deficits following viral infection [68], but may be related to deficits in the olfactory nerve or olfactory center. The other reason is that viruses can travel retrogradely along the cranial nerves into the brain and damage the nuclei of cranial nerves. It is therefore reasonable to speculate that olfactory impairment may be a sign of CNS damage following SARS-CoV-2 infection, but it remains unclear whether there is a definitive causal relationship between the two.

### OTHERS

#### Respiratory failure

Respiratory failure can be caused either by central respiratory pathology or by pulmonary pathology. It is therefore also worth considering whether the relationship between SARS-CoV-2 infection and damage to the nervous system is causal or coincidental. Many patients with severe SARS-CoV-2 infection develop respiratory failure [53, 69], which is an important cause of death. It is worth noting that respiratory failure can occur in addition to pulmonary causes, as well as damage to the respiratory center of the CNS. The question arises whether the CNS respiratory center involved in the development of respiratory failure in SARS-CoV-2 infected patients. It has been postulated that SARS-CoV-2 not only infects the lungs, but also has severe effects on neurons, especially those in the medulla oblongata that regulate respiratory, pulmonary, and cardiac functions, leading to respiratory failure [70]. It is therefore possible that some patients with SARS-CoV-2 develop respiratory failure despite no significant deterioration being visible in imaging examinations, and we should consider the possibility that SARS-CoV-2 may have directly invaded the respiratory center of the nervous system.

#### Headaches, dizziness, and muscle aches

Headaches, dizziness, and muscle aches can be symptoms of neurological injury as well as nonspecific injury symptoms due to respiratory and pulmonary infections. These symptoms often occur as nonspecific symptoms after SARS-CoV-2 infection. Nonspecific symptoms may act as a warning against possible neurological damage during the COVID-19 epidemic. Headaches and dizziness are the most common manifestations of SARS-CoV-2 infection and have been reported in many publications [7,71,72]. However, it is not clear whether headache and dizziness episodes are nonspecific symptoms of infection or are associated with neurological damage from SARS-CoV-2 infection. There are still no studies on the specific damage mechanisms of headache and dizziness in COVID-19 patients. Headaches occurred in 15.0% of the 1012 patients in the Wuhan Square Cabin Hospital in Hubei Province [73] and in 6.5% of 262 patients in Beijing [74], and in 34% of patients in a study in Zhejiang [75]. In Shanghai, dizziness and headache occurred in 11.2% of 249 patients [76]. Neurologists should therefore be wary of patients who attend the clinic with headaches and dizziness. Some patients present with the onset of syncope [77]. Others have no neurological symptoms but have positive neurological signs of neurological impairment, such as ataxia and pyramidal tract signs, on neurological examination [7,12]. Skeletal muscle damage is also a nonspecific symptom of SARS-CoV-2 infection. This could be related to direct viral attack leading to skeletal muscle lysis, and the immune response to viral infection. All of the above are nonspecific symptoms of the nervous system following SARS-CoV-2 infection. Whether these nonspecific symptoms are a manifestation of SARS-CoV-2 infection of the nervous system requires clarification in further studies.

### DISCUSSION

SARS-CoV-2 and the SARS coronavirus share genomic sequences which are similar, particularly the receptor-binding domain. Both enter the body through the angiotensin-converting enzyme 2 (ACE2) receptor [78]. This receptor is expressed in several organs and tissues of the body, including in the nervous system and skeletal muscle. This presents the possibility that SARS-CoV-2 may cause neurological damage through a direct or indirect mechanism [79].

Possible pathways for SARS-COV-2 to enter the CNS include either hematogenous or direct neuronal invasion [80]. The peripheral trigeminal nerve, olfactory nerve, or retina may be pathways by which the virus enters the CNS [81-83]. It has been suggested that SARS-CoV-2 can infect the intestinal mucosa, and even that SARS-CoV-2 reaches the CNS through the enteric nervous system and its sympathetic afferent neurons [80]. Once in the CNS, the virus can spread rapidly between neurons, leading to inflammation, degeneration, and even neuronal death.

While the exact mechanism by which SARS-CoV-2 infection causes damage to the CNS has not been specified, several possibilities have been proposed. First, the inflammatory response is an important cause. A few hours after infection, neutrophils and monocytes infiltrate the CNS, and neutrophils appear to be an important cause of disruptions to the blood-brain barrier (BBB) [84,85]. Immune cells then continue to infiltrate the brain in large numbers, which is associated with neuronal edema and degeneration [86]. Activated macrophages and microglia appear in the demyelinated region and play a key role in myelin destruction [85]. A large amount of myelin is immunogenic and reactivates macrophages following neuroinflammation, thus triggering a vicious cycle of inflammation. Secondly, SARS-CoV-2 directly infiltrates CNS by binding to ACE2 receptors on glial cells and neurons, where it causes a high level of secretion of inflammatory factors such as TNF-α, IL-1, etc., which in turn damage the nervous system [87-89].

In summary, whether the relationship between SARS-CoV-2 infection and neurological damage is causal or merely coincidental needs to be supported by data from large epidemiological studies and evidence from clinical follow-ups. Although our understanding of the mechanisms by which neurological damage occurs in SARS-CoV-2 infection is incomplete, we are actively exploring its possibilities. We hope that our report will be helpful to clinicians. To conclude, during the COVID-19 epidemic, neurologists should always first consider the possibility of SARS-CoV-2 infection in patients with neurological damage in the emergency department to avoid misdiagnosis or the spread of infection. Secondly, patients with SARS-CoV-2 infection can show symptoms of neurological damage, such as cerebrovascular disease, encephalitis, epilepsy, and demyelinating lesions. Clinicians should pay close attention to the signs of neurological damage in SARS-CoV-2-infected patients, which may lead to death, especially in patients with severe cases.
